# Solitary neurofibroma in the male breast

**DOI:** 10.1186/1477-7819-5-23

**Published:** 2007-02-27

**Authors:** Deva S Jeyaretna, Adewunmi Oriolowo, Mark EF Smith, Roger M Watkins

**Affiliations:** 1 Primrose Breast Care Centre, Derriford Hospital, Plymouth, UK; 2 Department of Histopathology, Derriford Hospital, Plymouth, UK

## Abstract

**Background:**

Neurofibroma of the male breast outside of neurofibromatosis is extremely rare with only one previous case having been reported.

**Case presentation:**

A 48 year old male patient with a neurofibroma in the breast presenting with gynaecomastia is reported. Clinical and mammogram findings with fine needle aspiration cytology and full histology are presented.

**Conclusion:**

To our knowledge this is only the second case of a neurofibroma in a male breast in the English literature and the first report to include the mammographic findings.

## Background

A patient with a neurofibroma in the breast presenting with gynaecomastia is reported. Neurofibroma of the male breast is extremely rare with only one previous case having been reported [1].

## Case presentation

A 48 year-old man presented with gynaecomastia, having first noticed a swelling of the left breast twenty years previously. This had gradually increased in size. Following an episode of recent weight loss the swelling had become more prominent.

On examination, he had a 4 × 4 cm firm mass which appeared to be attached to the skin and was situated immediately below the left nipple. There was no fixation to the underlying muscle and no lymphadenopathy. In addition, there were no features of neurofibromatosis and in particular there were no *café au lait *spots.

**Figure 1:**
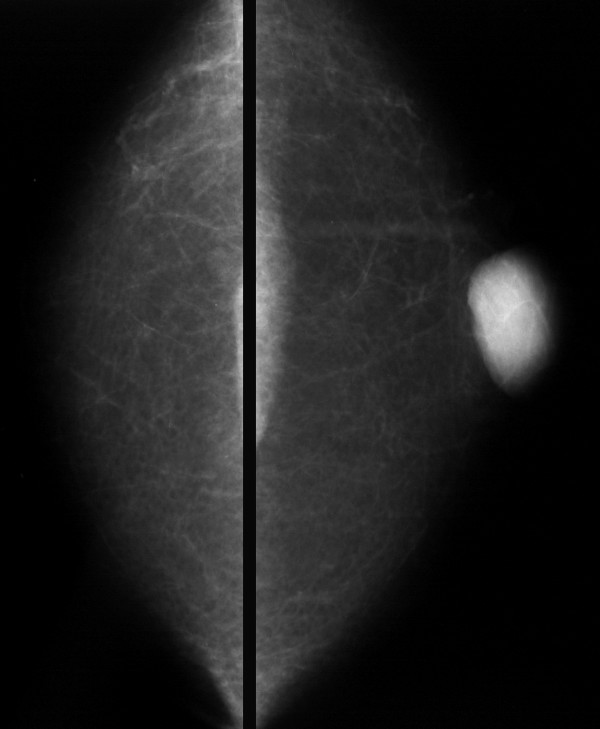
Cranio-caudal mammograms showing the mass lesion in the central area of the left breast.

Mammograms showed a well-defined mass measuring 36 mm in its maximum diameter and immediately adjacent to the left nipple (Figure 1). It was situated centrally within the breast tissue and immediately deep to the skin. The density of the mass was relatively low for its size.

Fine needle aspiration cytology showed stromal fragments containing spindle cells suggesting a soft tissue lesion of neural origin. Core needle biopsy revealed a spindle cell infiltrate. The spindle cells had irregular nuclei, many expressing S-100 protein. No mitoses were seen. Although a neurofibroma was suspected, several atypical features were present, including hyperchromasia of some nuclei, increased cellularity and the presence of relatively broad and long fasicles.

The tumour was excised under general anaesthetic with an ellipse of overlying skin but preserving the nipple areolar complex. The incision was an inferior periareolar incision to ensure optimum cosmesis. The overlying skin was taken because of the proximity of the tumour to the skin.

**Figure 2:**
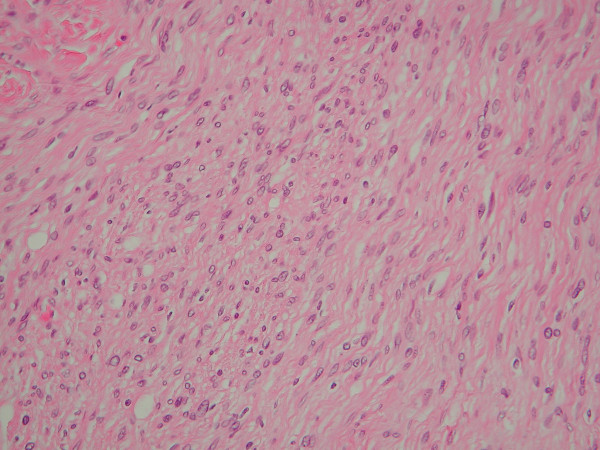
Photomicrograph of the resected neurofibroma showing spindle cells with characteristic elongated, wavy nuclei. (20× magnification, Haematoxylin and Eosin)

Macroscopically the tumour measured 4 × 3 × 2.5 cm, was white and well circumscribed. Microscopically it was moderately cellular and it contained spindle cells with irregular and focally pleomorphic nuclei. No mitoses or necrosis were seen (Figure 2). No Antoni A areas were present. A definitive histological diagnosis of a benign cellular neurofibroma was made. After 5-years no recurrence has been observed.

## Discussion

Neurofibromas are benign nerve sheath tumours which were first described by Smith in 1849 and later by von Recklinghausen in 1882 [2]. They are relatively common tumours with an equal incidence in both sexes and can occur at any age. The majority of neurofibromas are solitary lesions that occur in the dermis or subcutis and are evenly distributed over the body surface [3,4]. Their presence in the skin is more common than in the deeper soft tissues. Those occurring below the skin are usually in an axial distribution [4]. They are slow growing and the majority painless.

Clinically, several forms of neurofibroma are seen, including plexiform neurofibromas, diffuse neurofibromas and visceral neurofibromas. Solitary neurofibromas of the breast are rare, even in women where only four cases have been reported [2,5,6]. In the male breast, there has been only one previous case [1]. This excludes the occurrence of neurofibromas in the presence of neurofibromatosis (von Recklinghausen's disease) which is regarded as a separate disease process.

Macroscopically, neurofibromas are well circumscribed and if still confined by the epineurium are encapsulated. Most are not encapsulated. They vary in size and shape but most are between 1 cm and 2 cm [7]. Typically they are white-grey tumours as in the current case, but some are brown. They may be polypoid or fusiform in shape [7].

Histologically neurofibromas contain interlacing bundles of elongated cells with wavy, dark staining nuclei and slender cytoplasmic processes [3,8]^.^These cells are arranged closely and are separated by small amounts of mucoid material. Neurofibromas lack epithelial elements. They demonstrate S-100 positivity, typically in some but not all of their component spindle cells. In keeping with their benign behaviour they lack significant mitotic activity.

In present case mammograms demonstrated a homogeneous mass with a regular, well-demarcated border. There was no calcification or distortion of the surrounding breast architecture. These findings are as expected for a benign lesion of the breast. The mammographic characteristics of male breast cancer and their differences to gynaecomastia are well documented [9].

The primary differential diagnosis for this tumour is a neurilemmoma but fibroadenoma, phyllodes tumour, malignant peripheral nerve sheath tumour and myofibroblastoma should all be considered. Neurilemmomas may be differentiated from neurofibromas by the presence of Antoni A and B areas, Verocay bodies and uniform staining for S-100 protein [7]. All these features were lacking in the current case.

Treatment of neurofibromas is by surgical excision. Care should be taken in placing the incision and an inferior periareolar incision is preferred [10]. Solitary neurofibromas in general are associated with a low local recurrence rate if completely excised [7]. In none of the previously reported cases of a neurofibroma in the breast, has recurrence occurred.

## Conclusion

To our knowledge this is only the second case of a neurofibroma in a male breast in the English literature and the first report to include the mammographic findings. There are no reports of recurrence after complete surgical excision.

## Competing interests

The author(s) declare that we have no competing interests.

## Authors' contributions

**DSJ **and **RMW **designed the study, carried out data and picture acquisition and drafted the manuscript. **AO **and **MS **performed the histological assessments. All authors participated in the editing and have read and approved the final manuscript.
